# 2-Anilino-3-(2-hy­droxy­phen­yl)quinazolin-4(3*H*)-one–triphenyl­phosphine oxide (1/1)

**DOI:** 10.1107/S1600536810035324

**Published:** 2010-09-08

**Authors:** Hong-Ling Wang, Xiao-Bao Chen, Xu-Hong Yang, Dong-Feng Pan, Jun-Kai Ma

**Affiliations:** a Faculty of Chemistry and Life Science, Xianning University, Xianning 437100, Hubei, People’s Republic of China; b Institute of Medicinal Chemistry, Hubei Medical University, Shiyan 442000, Hubei, People’s Republic of China; c Department of Oncology, Renmin Hospital, Hubei Medical University, Shiyan 442000, Hubei, People’s Republic of China

## Abstract

In the title compound, C_20_H_15_N_3_O_2_·C_18_H_15_OP, the pyrimidinone heterocycle and the fused phenyl ring are inclined at 1.92 (7)°. Only the hy­droxy group is involved in hydrogen bonding, whereas the amino group is shielded from potential acceptors.

## Related literature

For the synthesis of the title compound, see: Yang *et al.* (2008 ▶).
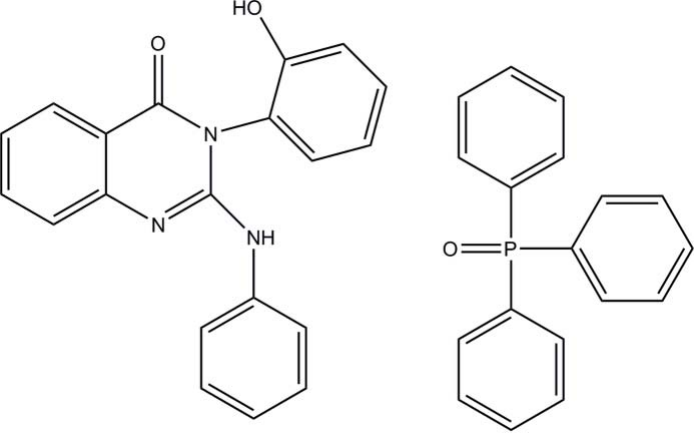


## Experimental

### 

#### Crystal data

C_20_H_15_N_3_O_2_·C_18_H_15_OP
*M* *_r_* = 607.62
Monoclinic, 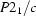

*a* = 18.9139 (3) Å
*b* = 10.3201 (2) Å
*c* = 18.2145 (3) Å
β = 117.771 (1)°
*V* = 3145.83 (9) Å^3^
*Z* = 4
Mo *K*α radiation
μ = 0.13 mm^−1^
*T* = 298 K
0.16 × 0.12 × 0.10 mm

#### Data collection

Bruker SMART APEX CCD area-detector diffractometer
Absorption correction: multi-scan (*SADABS*; Sheldrick, 2001 ▶) *T*_min_ = 0.980, *T*_max_ = 0.987
38085 measured reflections
7826 independent reflections
5767 reflections with *I* > 2σ(*I*)
*R*_int_ = 0.036

#### Refinement

*R*[*F* ^2^ > 2σ(*F* ^2^)] = 0.047
*wR*(*F* ^2^) = 0.134
*S* = 1.01
7826 reflections
412 parameters
H atoms treated by a mixture of independent and constrained refinement
Δρ_max_ = 0.37 e Å^−3^
Δρ_min_ = −0.23 e Å^−3^

### 

Data collection: *SMART* (Bruker, 2000 ▶); cell refinement: *SAINT* (Bruker, 2000 ▶); data reduction: *SAINT*; program(s) used to solve structure: *SHELXS97* (Sheldrick, 2008 ▶); program(s) used to refine structure: *SHELXL97* (Sheldrick, 2008 ▶); molecular graphics: *SHELXTL* (Sheldrick, 2008 ▶); software used to prepare material for publication: *SHELXTL* .

## Supplementary Material

Crystal structure: contains datablocks global, I. DOI: 10.1107/S1600536810035324/bt5345sup1.cif

Structure factors: contains datablocks I. DOI: 10.1107/S1600536810035324/bt5345Isup2.hkl

Additional supplementary materials:  crystallographic information; 3D view; checkCIF report

## Figures and Tables

**Table 1:** Hydrogen-bond geometry (Å, °)

*D*—H⋯*A*	*D*—H	H⋯*A*	*D*⋯*A*	*D*—H⋯*A*
O2—H2*A*⋯O3^i^	0.815 (18)	1.862 (19)	2.6436 (15)	160.1 (18)

Symmetry code: (i) 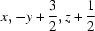.

**Fig. 1.**
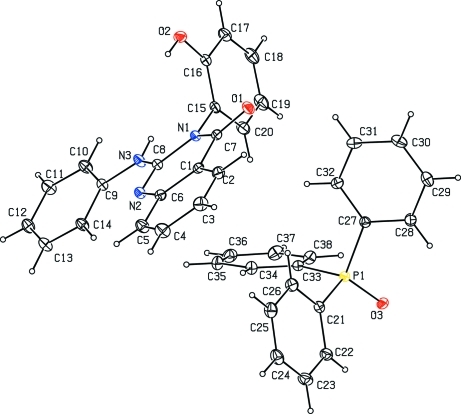
View of the molecular structure of the title compound, showing the atom labelling schemeand with displacement ellipsoids drawn at the 50% probability level.

## supplementary crystallographic information

### Comment

Quinazoline-4(3*H*)-one derivatives have numerous biological properties. We have recently focused on the synthesis of heterocyclic compounds using an aza-Wittig reaction. We present here the crystal structure of the title compound    (Fig. 1), which can be used as a precursor for obtaining bioactive molecules.

In the crystal structure, the pyrimidinone heterocycle and the adjacent benzene ring are not coplanar, but inclined at 1.92 (7) °. Only the hydroxyl group is involved in hydrogen bonding, whereas the amino group is shielded from potential acceptors.

### Experimental

To a solution of iminophosphorane (1.40 g, 3.0 mmol) in anhydrous THF (10 ml) was added isocyanatobenzene (3 mmol) under nitrogen at room temperature. After reaction, the mixture was allowed to stand for 10 h at 273–278 K, the solvent was removed under reduced pressure and diethyl ether/petroleum ether (1:2 *v*/*v*, 20 ml) was added to precipitate triphenylphosphine oxide. After filtration, the solvent was removed to give 1-phenyl- 3-(2-ethoxycarbonylphenyl) carbodiimide, which was used directly without further purification. To a solution of 1-phenyl- 3-(2-ethoxycarbonylphenyl) carbodiimide in THF (15 ml) was added 2-aminophenol (3 mmol). After the reaction mixture was allowed to stand for 0.5 h, the solvent was removed and anhydrous ethanol (10 ml) with several drops of EtONa in EtOH was added. The mixture was stirred for 2 h at room temperature. The solution was concentrated under reduced pressure and the residue was recrystallized from ethanol to give the title compound  (yield 79%).

### Refinement

All the carbon-bonded hydrogen atoms set to ideal positons with C—H = 0.93Å (aromatic) and 0.96Å (methyl), and *U*_iso_(H) = 1.2*U*_eq_C for aromatic and 1.5 *U*_eq_C for methyl hydrogen atoms, respectively. H atoms bonded to N and O atoms were found in a difference map and then refined with distance restraints of N—H = 0.85 (2)Å and O—H = 0.90 (2) Å. The displacement parameters were set *U*_iso_(H) = 1.2*U*_eq_N or *U*_iso_(H) = 1.5*U*_eq_O.

### Crystal data

**Table:**

C_20_H_15_N_3_O_2_·C_18_H_15_OP	*F*(000) = 1272
*M**_r_* = 607.62	*D*_x_ = 1.283 Mg m^−^^3^
Monoclinic, *P*2_1_/*c*	Mo *K*α radiation, λ = 0.71073 Å
*a* = 18.9139 (3) Å	Cell parameters from 5201 reflections
*b* = 10.3201 (2) Å	θ = 2.3–26.1°
*c* = 18.2145 (3) Å	µ = 0.13 mm^−^^1^
β = 117.771 (1)°	*T* = 298 K
*V* = 3145.83 (9) Å^3^	Block, colorless
*Z* = 4	0.16 × 0.12 × 0.10 mm

### Data collection

**Table:**

Bruker SMART APEX CCD area-detector diffractometer	7826 independent reflections
Radiation source: fine-focus sealed tube	5767 reflections with *I* > 2σ(*I*)
graphite	*R*_int_ = 0.036
φ and ω scans	θ_max_ = 28.3°, θ_min_ = 2.2°
Absorption correction: multi-scan (*SADABS*; Sheldrick, 2001)	*h* = −25→25
*T*_min_ = 0.980, *T*_max_ = 0.987	*k* = −13→13
38085 measured reflections	*l* = −24→24

### Refinement

**Table:**

Refinement on *F*^2^	Primary atom site location: structure-invariant direct methods
Least-squares matrix: full	Secondary atom site location: difference Fourier map
*R*[*F*^2^ > 2σ(*F*^2^)] = 0.047	Hydrogen site location: inferred from neighbouring sites
*wR*(*F*^2^) = 0.134	H atoms treated by a mixture of independent and constrained refinement
*S* = 1.01	*w* = 1/[σ^2^(*F*_o_^2^) + (0.0807*P*)^2^] where *P* = (*F*_o_^2^ + 2*F*_c_^2^)/3
7826 reflections	(Δ/σ)_max_ = 0.001
412 parameters	Δρ_max_ = 0.37 e Å^−^^3^
0 restraints	Δρ_min_ = −0.23 e Å^−^^3^

### Special details

**Table:**

Geometry. All e.s.d.'s (except the e.s.d. in the dihedral angle between two l.s. planes) are estimated using the full covariance matrix. The cell e.s.d.'s are taken into account individually in the estimation of e.s.d.'s in distances, angles and torsion angles; correlations between e.s.d.'s in cell parameters are only used when they are defined by crystal symmetry. An approximate (isotropic) treatment of cell e.s.d.'s is used for estimating e.s.d.'s involving l.s. planes.
Refinement. Refinement of *F*^2^ against ALL reflections. The weighted *R*-factor *wR* and goodness of fit *S* are based on *F*^2^, conventional *R*-factors *R* are based on *F*, with *F* set to zero for negative *F*^2^. The threshold expression of *F*^2^ > σ(*F*^2^) is used only for calculating *R*-factors(gt) *etc*. and is not relevant to the choice of reflections for refinement. *R*-factors based on *F*^2^ are statistically about twice as large as those based on *F*, and *R*- factors based on ALL data will be even larger.

### Fractional atomic coordinates and isotropic or equivalent isotropic displacement parameters (Å^2^)

**Table:**

	*x*	*y*	*z*	*U*_iso_*/*U*_eq_	
C1	0.32962 (8)	0.72741 (14)	0.53651 (9)	0.0427 (3)	
C2	0.40582 (9)	0.67312 (16)	0.56734 (10)	0.0553 (4)	
H2	0.4466	0.7194	0.5644	0.066*	
C3	0.42037 (10)	0.55219 (17)	0.60173 (12)	0.0648 (5)	
H3	0.4710	0.5160	0.6219	0.078*	
C4	0.35995 (10)	0.48339 (18)	0.60672 (12)	0.0698 (5)	
H4	0.3703	0.4012	0.6303	0.084*	
C5	0.28496 (10)	0.53564 (16)	0.57706 (11)	0.0602 (4)	
H5	0.2450	0.4891	0.5815	0.072*	
C6	0.26804 (8)	0.65830 (13)	0.54015 (9)	0.0438 (3)	
C7	0.31351 (8)	0.85644 (13)	0.50056 (9)	0.0431 (3)	
C8	0.17650 (8)	0.81807 (13)	0.47432 (9)	0.0407 (3)	
C9	0.03094 (8)	0.81895 (13)	0.43622 (9)	0.0426 (3)	
C10	−0.03180 (9)	0.90485 (15)	0.41509 (11)	0.0573 (4)	
H10	−0.0256	0.9911	0.4044	0.069*	
C11	−0.10340 (9)	0.86384 (17)	0.40972 (12)	0.0651 (5)	
H11	−0.1451	0.9225	0.3953	0.078*	
C12	−0.11359 (9)	0.73755 (17)	0.42540 (11)	0.0588 (4)	
H12	−0.1614	0.7104	0.4233	0.071*	
C13	−0.05256 (9)	0.65174 (16)	0.44417 (11)	0.0579 (4)	
H13	−0.0599	0.5653	0.4534	0.069*	
C14	0.01986 (9)	0.69020 (14)	0.44979 (10)	0.0533 (4)	
H14	0.0607	0.6303	0.4625	0.064*	
C15	0.21124 (8)	1.01666 (14)	0.42224 (9)	0.0435 (3)	
C16	0.22674 (8)	1.13421 (14)	0.46389 (9)	0.0444 (3)	
C17	0.20183 (10)	1.24752 (16)	0.41711 (11)	0.0595 (4)	
H17	0.2115	1.3273	0.4439	0.071*	
C18	0.16332 (11)	1.2428 (2)	0.33218 (12)	0.0701 (5)	
H18	0.1467	1.3194	0.3020	0.084*	
C19	0.14897 (11)	1.1270 (2)	0.29119 (11)	0.0762 (6)	
H19	0.1229	1.1247	0.2335	0.091*	
C20	0.17354 (10)	1.01345 (18)	0.33622 (10)	0.0642 (5)	
H20	0.1648	0.9345	0.3087	0.077*	
C21	0.33743 (8)	0.43284 (13)	0.30104 (9)	0.0433 (3)	
C22	0.32238 (9)	0.31200 (15)	0.26340 (11)	0.0537 (4)	
H22	0.2973	0.3058	0.2058	0.064*	
C23	0.34463 (11)	0.20058 (16)	0.31131 (13)	0.0675 (5)	
H23	0.3350	0.1199	0.2858	0.081*	
C24	0.38093 (11)	0.20870 (18)	0.39657 (13)	0.0674 (5)	
H24	0.3944	0.1337	0.4285	0.081*	
C25	0.39715 (11)	0.32773 (18)	0.43414 (11)	0.0649 (5)	
H25	0.4224	0.3332	0.4917	0.078*	
C26	0.37629 (10)	0.43949 (16)	0.38725 (10)	0.0554 (4)	
H26	0.3883	0.5198	0.4134	0.066*	
C27	0.37978 (8)	0.69513 (13)	0.28300 (8)	0.0409 (3)	
C28	0.43786 (9)	0.70114 (15)	0.25692 (10)	0.0523 (4)	
H28	0.4348	0.6466	0.2149	0.063*	
C29	0.50034 (10)	0.78835 (18)	0.29339 (11)	0.0636 (5)	
H29	0.5388	0.7929	0.2753	0.076*	
C30	0.50577 (10)	0.86780 (17)	0.35592 (12)	0.0649 (5)	
H30	0.5483	0.9253	0.3807	0.078*	
C31	0.44867 (11)	0.86288 (16)	0.38213 (11)	0.0642 (5)	
H31	0.4525	0.9170	0.4246	0.077*	
C32	0.38546 (10)	0.77778 (15)	0.34559 (10)	0.0542 (4)	
H32	0.3464	0.7758	0.3630	0.065*	
C33	0.21504 (8)	0.63051 (13)	0.24196 (9)	0.0435 (3)	
C34	0.19543 (10)	0.59243 (16)	0.30366 (10)	0.0560 (4)	
H34	0.2309	0.5414	0.3475	0.067*	
C35	0.12366 (11)	0.62987 (19)	0.30023 (12)	0.0677 (5)	
H35	0.1111	0.6040	0.3417	0.081*	
C36	0.07094 (11)	0.70508 (19)	0.23571 (13)	0.0703 (5)	
H36	0.0225	0.7292	0.2331	0.084*	
C37	0.08972 (11)	0.74456 (19)	0.17519 (12)	0.0687 (5)	
H37	0.0541	0.7962	0.1319	0.082*	
C38	0.16126 (10)	0.70831 (16)	0.17799 (10)	0.0566 (4)	
H38	0.1735	0.7362	0.1367	0.068*	
N1	0.23328 (6)	0.89554 (11)	0.46688 (7)	0.0402 (3)	
N2	0.19109 (7)	0.70555 (11)	0.50893 (7)	0.0443 (3)	
H2A	0.2741 (11)	1.0768 (17)	0.5709 (11)	0.066*	
N3	0.10191 (7)	0.87046 (12)	0.44097 (9)	0.0511 (3)	
H3A	0.0993 (10)	0.9481 (17)	0.4256 (10)	0.061*	
O1	0.36237 (6)	0.92844 (10)	0.49659 (7)	0.0590 (3)	
O2	0.26529 (7)	1.14642 (10)	0.54705 (7)	0.0558 (3)	
O3	0.28491 (6)	0.54813 (10)	0.14922 (6)	0.0496 (3)	
P1	0.30304 (2)	0.57484 (3)	0.23650 (2)	0.03965 (11)	

### Atomic displacement parameters (Å^2^)

**Table:**

	*U*^11^	*U*^22^	*U*^33^	*U*^12^	*U*^13^	*U*^23^
C1	0.0443 (7)	0.0437 (7)	0.0428 (7)	−0.0029 (6)	0.0226 (6)	−0.0082 (6)
C2	0.0449 (8)	0.0609 (10)	0.0618 (10)	−0.0022 (7)	0.0263 (7)	−0.0098 (8)
C3	0.0482 (9)	0.0657 (11)	0.0748 (12)	0.0127 (8)	0.0239 (8)	0.0008 (9)
C4	0.0644 (11)	0.0566 (10)	0.0835 (13)	0.0147 (9)	0.0302 (10)	0.0152 (9)
C5	0.0540 (9)	0.0507 (9)	0.0759 (12)	0.0026 (7)	0.0303 (8)	0.0111 (8)
C6	0.0442 (8)	0.0423 (7)	0.0468 (8)	−0.0011 (6)	0.0226 (6)	−0.0024 (6)
C7	0.0445 (7)	0.0458 (8)	0.0445 (8)	−0.0081 (6)	0.0254 (6)	−0.0107 (6)
C8	0.0429 (7)	0.0388 (7)	0.0438 (7)	−0.0043 (6)	0.0232 (6)	−0.0007 (6)
C9	0.0388 (7)	0.0432 (7)	0.0446 (8)	−0.0023 (6)	0.0184 (6)	0.0051 (6)
C10	0.0484 (9)	0.0459 (8)	0.0730 (11)	0.0036 (7)	0.0244 (8)	0.0139 (7)
C11	0.0433 (9)	0.0594 (10)	0.0887 (13)	0.0088 (7)	0.0275 (9)	0.0123 (9)
C12	0.0394 (8)	0.0653 (10)	0.0691 (11)	−0.0025 (7)	0.0232 (7)	0.0095 (8)
C13	0.0445 (8)	0.0512 (9)	0.0728 (11)	−0.0035 (7)	0.0230 (8)	0.0146 (8)
C14	0.0440 (8)	0.0436 (8)	0.0712 (11)	0.0032 (6)	0.0259 (8)	0.0116 (7)
C15	0.0447 (7)	0.0448 (8)	0.0429 (8)	−0.0083 (6)	0.0221 (6)	0.0052 (6)
C16	0.0456 (8)	0.0432 (7)	0.0483 (8)	−0.0051 (6)	0.0251 (7)	0.0046 (6)
C17	0.0643 (10)	0.0459 (9)	0.0711 (11)	−0.0018 (7)	0.0339 (9)	0.0121 (8)
C18	0.0636 (11)	0.0694 (12)	0.0682 (12)	−0.0042 (9)	0.0230 (9)	0.0301 (10)
C19	0.0767 (12)	0.0890 (14)	0.0466 (10)	−0.0218 (11)	0.0150 (9)	0.0193 (10)
C20	0.0740 (11)	0.0667 (11)	0.0454 (9)	−0.0251 (9)	0.0225 (8)	−0.0012 (8)
C21	0.0419 (7)	0.0429 (7)	0.0479 (8)	−0.0008 (6)	0.0234 (6)	0.0042 (6)
C22	0.0565 (9)	0.0459 (8)	0.0599 (10)	−0.0015 (7)	0.0280 (8)	0.0011 (7)
C23	0.0735 (12)	0.0426 (9)	0.0881 (14)	0.0002 (8)	0.0390 (10)	0.0045 (9)
C24	0.0691 (11)	0.0563 (10)	0.0846 (13)	0.0125 (9)	0.0425 (10)	0.0265 (10)
C25	0.0682 (11)	0.0696 (12)	0.0579 (10)	0.0128 (9)	0.0302 (9)	0.0193 (9)
C26	0.0599 (9)	0.0532 (9)	0.0508 (9)	0.0034 (7)	0.0239 (8)	0.0061 (7)
C27	0.0444 (7)	0.0383 (7)	0.0387 (7)	−0.0001 (6)	0.0183 (6)	0.0031 (6)
C28	0.0530 (9)	0.0566 (9)	0.0520 (9)	−0.0019 (7)	0.0284 (7)	0.0006 (7)
C29	0.0490 (9)	0.0720 (11)	0.0712 (11)	−0.0074 (8)	0.0290 (8)	0.0096 (9)
C30	0.0546 (10)	0.0534 (10)	0.0680 (11)	−0.0139 (8)	0.0129 (8)	0.0041 (8)
C31	0.0679 (11)	0.0516 (9)	0.0640 (11)	−0.0091 (8)	0.0229 (9)	−0.0147 (8)
C32	0.0574 (9)	0.0522 (9)	0.0566 (9)	−0.0057 (7)	0.0296 (8)	−0.0103 (7)
C33	0.0464 (8)	0.0419 (7)	0.0435 (8)	−0.0069 (6)	0.0221 (6)	−0.0068 (6)
C34	0.0592 (9)	0.0602 (10)	0.0554 (9)	−0.0022 (8)	0.0325 (8)	0.0006 (7)
C35	0.0703 (11)	0.0755 (12)	0.0774 (13)	−0.0112 (10)	0.0512 (10)	−0.0132 (10)
C36	0.0534 (10)	0.0749 (12)	0.0870 (14)	−0.0026 (9)	0.0364 (10)	−0.0225 (11)
C37	0.0588 (10)	0.0724 (12)	0.0669 (11)	0.0149 (9)	0.0227 (9)	−0.0037 (9)
C38	0.0591 (10)	0.0599 (10)	0.0519 (9)	0.0074 (8)	0.0268 (8)	0.0011 (7)
N1	0.0435 (6)	0.0385 (6)	0.0418 (6)	−0.0074 (5)	0.0226 (5)	−0.0012 (5)
N2	0.0430 (6)	0.0405 (6)	0.0512 (7)	−0.0007 (5)	0.0235 (6)	0.0052 (5)
N3	0.0448 (7)	0.0408 (7)	0.0697 (9)	0.0007 (5)	0.0283 (6)	0.0152 (6)
O1	0.0520 (6)	0.0544 (6)	0.0810 (8)	−0.0122 (5)	0.0397 (6)	−0.0026 (5)
O2	0.0792 (8)	0.0408 (6)	0.0485 (6)	−0.0031 (5)	0.0307 (6)	−0.0003 (5)
O3	0.0628 (6)	0.0483 (6)	0.0400 (6)	−0.0044 (5)	0.0258 (5)	−0.0055 (4)
P1	0.0454 (2)	0.0385 (2)	0.0372 (2)	−0.00294 (15)	0.02114 (16)	−0.00170 (14)

### Geometric parameters (Å, °)

**Table:**

C1—C6	1.3935 (19)	C20—H20	0.9300
C1—C2	1.397 (2)	C21—C22	1.387 (2)
C1—C7	1.452 (2)	C21—C26	1.391 (2)
C2—C3	1.366 (2)	C21—P1	1.8000 (14)
C2—H2	0.9300	C22—C23	1.385 (2)
C3—C4	1.384 (2)	C22—H22	0.9300
C3—H3	0.9300	C23—C24	1.377 (3)
C4—C5	1.371 (2)	C23—H23	0.9300
C4—H4	0.9300	C24—C25	1.370 (3)
C5—C6	1.399 (2)	C24—H24	0.9300
C5—H5	0.9300	C25—C26	1.379 (2)
C6—N2	1.3812 (17)	C25—H25	0.9300
C7—O1	1.2141 (16)	C26—H26	0.9300
C7—N1	1.4050 (17)	C27—C28	1.386 (2)
C8—N2	1.2884 (17)	C27—C32	1.387 (2)
C8—N3	1.3612 (18)	C27—P1	1.7944 (14)
C8—N1	1.3952 (16)	C28—C29	1.384 (2)
C9—C10	1.384 (2)	C28—H28	0.9300
C9—C14	1.3851 (19)	C29—C30	1.368 (3)
C9—N3	1.4084 (17)	C29—H29	0.9300
C10—C11	1.378 (2)	C30—C31	1.370 (3)
C10—H10	0.9300	C30—H30	0.9300
C11—C12	1.367 (2)	C31—C32	1.379 (2)
C11—H11	0.9300	C31—H31	0.9300
C12—C13	1.366 (2)	C32—H32	0.9300
C12—H12	0.9300	C33—C38	1.390 (2)
C13—C14	1.383 (2)	C33—C34	1.394 (2)
C13—H13	0.9300	C33—P1	1.8067 (15)
C14—H14	0.9300	C34—C35	1.385 (2)
C15—C20	1.387 (2)	C34—H34	0.9300
C15—C16	1.388 (2)	C35—C36	1.373 (3)
C15—N1	1.4428 (17)	C35—H35	0.9300
C16—O2	1.3463 (17)	C36—C37	1.368 (3)
C16—C17	1.393 (2)	C36—H36	0.9300
C17—C18	1.370 (2)	C37—C38	1.382 (2)
C17—H17	0.9300	C37—H37	0.9300
C18—C19	1.368 (3)	C38—H38	0.9300
C18—H18	0.9300	N3—H3A	0.843 (17)
C19—C20	1.381 (2)	O2—H2A	0.815 (18)
C19—H19	0.9300	O3—P1	1.4876 (10)
			
C6—C1—C2	120.27 (14)	C23—C22—H22	119.9
C6—C1—C7	119.10 (12)	C21—C22—H22	119.9
C2—C1—C7	120.62 (13)	C24—C23—C22	120.36 (16)
C3—C2—C1	120.03 (15)	C24—C23—H23	119.8
C3—C2—H2	120.0	C22—C23—H23	119.8
C1—C2—H2	120.0	C25—C24—C23	119.73 (16)
C2—C3—C4	120.19 (15)	C25—C24—H24	120.1
C2—C3—H3	119.9	C23—C24—H24	120.1
C4—C3—H3	119.9	C24—C25—C26	120.52 (17)
C5—C4—C3	120.44 (16)	C24—C25—H25	119.7
C5—C4—H4	119.8	C26—C25—H25	119.7
C3—C4—H4	119.8	C25—C26—C21	120.40 (16)
C4—C5—C6	120.55 (15)	C25—C26—H26	119.8
C4—C5—H5	119.7	C21—C26—H26	119.8
C6—C5—H5	119.7	C28—C27—C32	118.93 (14)
N2—C6—C1	122.91 (13)	C28—C27—P1	117.71 (11)
N2—C6—C5	118.60 (13)	C32—C27—P1	123.30 (11)
C1—C6—C5	118.49 (13)	C29—C28—C27	120.00 (15)
O1—C7—N1	119.70 (13)	C29—C28—H28	120.0
O1—C7—C1	125.62 (13)	C27—C28—H28	120.0
N1—C7—C1	114.66 (11)	C30—C29—C28	120.37 (16)
N2—C8—N3	121.17 (12)	C30—C29—H29	119.8
N2—C8—N1	124.40 (12)	C28—C29—H29	119.8
N3—C8—N1	114.44 (12)	C29—C30—C31	120.15 (15)
C10—C9—C14	118.85 (13)	C29—C30—H30	119.9
C10—C9—N3	116.38 (12)	C31—C30—H30	119.9
C14—C9—N3	124.76 (13)	C30—C31—C32	120.15 (16)
C11—C10—C9	120.65 (15)	C30—C31—H31	119.9
C11—C10—H10	119.7	C32—C31—H31	119.9
C9—C10—H10	119.7	C31—C32—C27	120.38 (15)
C12—C11—C10	120.49 (15)	C31—C32—H32	119.8
C12—C11—H11	119.8	C27—C32—H32	119.8
C10—C11—H11	119.8	C38—C33—C34	118.22 (15)
C13—C12—C11	119.05 (15)	C38—C33—P1	118.08 (11)
C13—C12—H12	120.5	C34—C33—P1	123.54 (12)
C11—C12—H12	120.5	C35—C34—C33	120.56 (17)
C12—C13—C14	121.64 (15)	C35—C34—H34	119.7
C12—C13—H13	119.2	C33—C34—H34	119.7
C14—C13—H13	119.2	C36—C35—C34	120.14 (17)
C13—C14—C9	119.27 (14)	C36—C35—H35	119.9
C13—C14—H14	120.4	C34—C35—H35	119.9
C9—C14—H14	120.4	C37—C36—C35	120.01 (17)
C20—C15—C16	120.33 (14)	C37—C36—H36	120.0
C20—C15—N1	118.52 (13)	C35—C36—H36	120.0
C16—C15—N1	121.15 (12)	C36—C37—C38	120.46 (18)
O2—C16—C15	124.23 (13)	C36—C37—H37	119.8
O2—C16—C17	117.46 (14)	C38—C37—H37	119.8
C15—C16—C17	118.31 (14)	C37—C38—C33	120.59 (16)
C18—C17—C16	120.74 (16)	C37—C38—H38	119.7
C18—C17—H17	119.6	C33—C38—H38	119.7
C16—C17—H17	119.6	C8—N1—C7	121.11 (11)
C19—C18—C17	120.90 (16)	C8—N1—C15	121.18 (11)
C19—C18—H18	119.5	C7—N1—C15	117.69 (11)
C17—C18—H18	119.5	C8—N2—C6	117.65 (12)
C18—C19—C20	119.40 (17)	C8—N3—C9	129.60 (12)
C18—C19—H19	120.3	C8—N3—H3A	114.5 (11)
C20—C19—H19	120.3	C9—N3—H3A	115.5 (11)
C19—C20—C15	120.29 (17)	C16—O2—H2A	112.7 (13)
C19—C20—H20	119.9	O3—P1—C27	111.44 (6)
C15—C20—H20	119.9	O3—P1—C21	112.35 (6)
C22—C21—C26	118.76 (14)	C27—P1—C21	106.76 (6)
C22—C21—P1	118.73 (12)	O3—P1—C33	110.99 (6)
C26—C21—P1	122.49 (11)	C27—P1—C33	108.51 (6)
C23—C22—C21	120.18 (16)	C21—P1—C33	106.55 (7)
			
C6—C1—C2—C3	−0.4 (2)	C28—C27—C32—C31	−1.1 (2)
C7—C1—C2—C3	179.35 (14)	P1—C27—C32—C31	176.10 (12)
C1—C2—C3—C4	−0.5 (3)	C38—C33—C34—C35	1.0 (2)
C2—C3—C4—C5	0.2 (3)	P1—C33—C34—C35	−174.42 (13)
C3—C4—C5—C6	1.0 (3)	C33—C34—C35—C36	0.0 (3)
C2—C1—C6—N2	−178.41 (13)	C34—C35—C36—C37	−0.8 (3)
C7—C1—C6—N2	1.8 (2)	C35—C36—C37—C38	0.6 (3)
C2—C1—C6—C5	1.6 (2)	C36—C37—C38—C33	0.4 (3)
C7—C1—C6—C5	−178.21 (14)	C34—C33—C38—C37	−1.2 (2)
C4—C5—C6—N2	178.11 (15)	P1—C33—C38—C37	174.44 (13)
C4—C5—C6—C1	−1.9 (2)	N2—C8—N1—C7	−2.4 (2)
C6—C1—C7—O1	176.98 (14)	N3—C8—N1—C7	178.00 (12)
C2—C1—C7—O1	−2.8 (2)	N2—C8—N1—C15	175.68 (13)
C6—C1—C7—N1	−4.37 (18)	N3—C8—N1—C15	−3.87 (18)
C2—C1—C7—N1	175.84 (12)	O1—C7—N1—C8	−176.54 (12)
C14—C9—C10—C11	1.7 (3)	C1—C7—N1—C8	4.71 (18)
N3—C9—C10—C11	−179.32 (15)	O1—C7—N1—C15	5.26 (19)
C9—C10—C11—C12	0.2 (3)	C1—C7—N1—C15	−173.48 (11)
C10—C11—C12—C13	−1.8 (3)	C20—C15—N1—C8	−77.32 (17)
C11—C12—C13—C14	1.7 (3)	C16—C15—N1—C8	102.31 (15)
C12—C13—C14—C9	0.1 (3)	C20—C15—N1—C7	100.87 (15)
C10—C9—C14—C13	−1.8 (2)	C16—C15—N1—C7	−79.50 (16)
N3—C9—C14—C13	179.28 (15)	N3—C8—N2—C6	179.02 (13)
C20—C15—C16—O2	−177.73 (14)	N1—C8—N2—C6	−0.5 (2)
N1—C15—C16—O2	2.6 (2)	C1—C6—N2—C8	0.8 (2)
C20—C15—C16—C17	1.5 (2)	C5—C6—N2—C8	−179.21 (14)
N1—C15—C16—C17	−178.15 (13)	N2—C8—N3—C9	−0.7 (2)
O2—C16—C17—C18	179.01 (14)	N1—C8—N3—C9	178.85 (14)
C15—C16—C17—C18	−0.3 (2)	C10—C9—N3—C8	166.44 (15)
C16—C17—C18—C19	−0.6 (3)	C14—C9—N3—C8	−14.6 (3)
C17—C18—C19—C20	0.2 (3)	C28—C27—P1—O3	−31.45 (13)
C18—C19—C20—C15	1.1 (3)	C32—C27—P1—O3	151.32 (12)
C16—C15—C20—C19	−1.9 (2)	C28—C27—P1—C21	91.57 (12)
N1—C15—C20—C19	177.73 (14)	C32—C27—P1—C21	−85.66 (14)
C26—C21—C22—C23	1.1 (2)	C28—C27—P1—C33	−153.95 (11)
P1—C21—C22—C23	−176.91 (12)	C32—C27—P1—C33	28.83 (15)
C21—C22—C23—C24	0.8 (3)	C22—C21—P1—O3	−18.40 (14)
C22—C23—C24—C25	−1.9 (3)	C26—C21—P1—O3	163.67 (11)
C23—C24—C25—C26	1.0 (3)	C22—C21—P1—C27	−140.85 (12)
C24—C25—C26—C21	0.9 (3)	C26—C21—P1—C27	41.22 (14)
C22—C21—C26—C25	−2.0 (2)	C22—C21—P1—C33	103.34 (12)
P1—C21—C26—C25	175.96 (12)	C26—C21—P1—C33	−74.59 (13)
C32—C27—C28—C29	0.2 (2)	C38—C33—P1—O3	−35.40 (14)
P1—C27—C28—C29	−177.17 (12)	C34—C33—P1—O3	139.99 (12)
C27—C28—C29—C30	0.8 (2)	C38—C33—P1—C27	87.37 (13)
C28—C29—C30—C31	−0.9 (3)	C34—C33—P1—C27	−97.24 (13)
C29—C30—C31—C32	0.0 (3)	C38—C33—P1—C21	−158.00 (12)
C30—C31—C32—C27	1.0 (3)	C34—C33—P1—C21	17.38 (14)

### Hydrogen-bond geometry (Å, °)

**Table:**

*D*—H···*A*	*D*—H	H···*A*	*D*···*A*	*D*—H···*A*
O2—H2A···O3^i^	0.815 (18)	1.862 (19)	2.6436 (15)	160.1 (18)

Symmetry codes: (i) *x*, −*y*+3/2, *z*+1/2.
